# Iliac Pseudoaneurysm from Endoleak

**DOI:** 10.5811/westjem.2015.8.28284

**Published:** 2015-12-01

**Authors:** Peter Moffett, Travis Redmon, Michael J. Vitto, David Evans

**Affiliations:** Virginia Commonwealth University Health System, Department of Emergency Medicine, Richmond, Virginia

A 65-year-old male presented to the emergency department complaining of two hours of severe lower abdominal pain radiating into his left testicle. The patient described a vascular procedure in the past but did not recall the details. An emergent bedside ultrasound was performed to evaluate the abdominal aorta. During the exam an echogenic object consistent with a prior endovascular stent was discovered in the distal aorta prompting further ultrasound evaluation of the iliac artery (Figure). A true lumen (thin black arrow) was visualized with evidence of leak (white arrows) during color Doppler evaluation. The patient was taken emergently to computed tomography and the diagnosis of an iliac artery pseudoaneurysm from an endoleak was confirmed.

A pseudoaneurysm is formed after a disruption causes a saccular expansion at the site of injury that is contained by adventitia or perivascular soft tissue. Rupture is common in patients with iliac artery pseudoaneurysm, with associated mortality rates of approximately 50%.^1^ An endoleak is a potential complication of endovascular stenting that involves blood leaking around or through the graft site.^2^

Presenting symptoms of a pseudoaneurysm are variable, based on the location, and are often caused by pressure on adjacent organs. Symptoms that have been described include abdominal pain, urinary symptoms, renal failure, lumbosacral pain, groin pain, rectal bleeding, or constipation.^1^

Our patient had prior endovascular stenting of an iliac artery aneurysm that extended into the distal aorta. He had developed a pseudoaneurysm (thick black arrow) arising from the medial aspect of the left iliac artery at the juncture of two metallic stents with active extravasation suggestive of an endoleak (white arrows). The patient underwent endovascular repair with endograft placement to repair the leak and subsequent coil embolization of the pseudoaneurysm cavity.

## Figures and Tables

**Figure:**
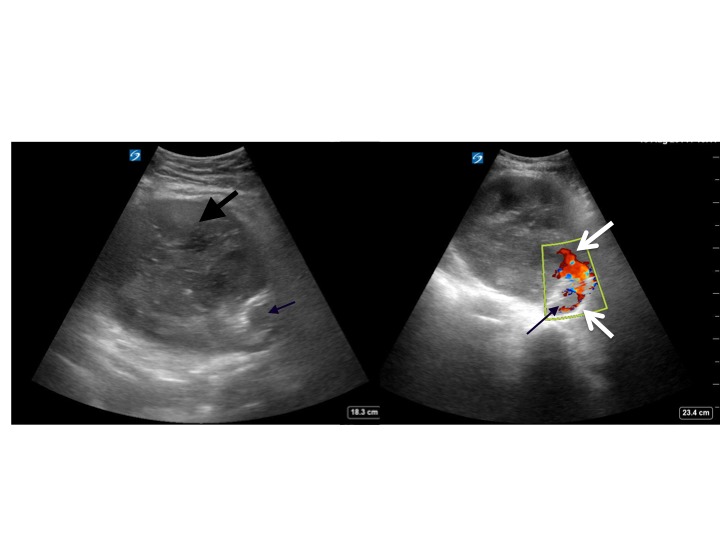
Ultrasound of the left iliac (left) with color Doppler flow (right) showing the true lumen (thin black arrow) and evidence of the leak (white arrows) creating a pseudoaneurysm (thick black arrow).
